# Bioengineered Nanoparticles Loaded-Hydrogels to Target TNF Alpha in Inflammatory Diseases

**DOI:** 10.3390/pharmaceutics13081111

**Published:** 2021-07-21

**Authors:** Isabel Matos Oliveira, Diogo Castro Fernandes, Fátima Raquel Maia, Raphael Faustino Canadas, Rui Luís Reis, Joaquim Miguel Oliveira

**Affiliations:** 1 13B’s Research Group, I3Bs—Research Institute on Biomaterials, Biodegradables and Biomimetics of University of Minho, Headquarters of the European Institute of Excellence on Tissue Engineering and Regenerative Medicine, Avepark, Parque de Ciência e Tecnologia, Zona Industrial da Gandra, 4805-017 Barco, Guimarães, Portugal; isabel2oliveira@hotmail.com (I.M.O.); diogoraposofernandes@gmail.com (D.C.F.); raquel.maia@i3bs.uminho.pt (F.R.M.); raphaelcanadas@gmail.com (R.F.C.); rgreis@i3bs.uminho.pt (R.L.R.); 2 ICVS/3B’s—PT Government Associate Laboratory, 4710-057 Braga, Braga, Portugal

**Keywords:** dendrimers, nanocomposite hydrogels, therapeutic efficacy, static conditions, dynamic conditions, bioreactor

## Abstract

Rheumatoid Arthritis (RA) is an incurable autoimmune disease that promotes the chronic impairment of patients’ mobility. For this reason, it is vital to develop therapies that target early inflammatory symptoms and act before permanent articular damage. The present study offers two novel therapies based in advanced drug delivery systems for RA treatment: encapsulated chondroitin sulfate modified poly(amidoamine) dendrimer nanoparticles (NPs) covalently bonded to monoclonal anti-TNF α antibody in both Tyramine-Gellan Gum and Tyramine-Gellan Gum/Silk Fibroin hydrogels. Using pro-inflammatory THP-1 (i.e., human monocytic cell line), the therapy was tested in an inflammation in vitro model under both static and dynamic conditions. Firstly, we demonstrated effective NP-antibody functionalization and TNF-α capture. Upon encapsulation, the NPs were released steadily over 21 days. Moreover, in static conditions, the approaches presented good anti-inflammatory activity over time, enabling the retainment of a high percentage of TNF α. To mimic the physiological conditions of the human body, the hydrogels were evaluated in a dual-chamber bioreactor. Dynamic in vitro studies showed absent cytotoxicity in THP-1 cells and a significant reduction of TNF-α in suspension over 14 days for both hydrogels. Thus, the developed approach showed potential for use as personalized medicine to obtain better therapeutic outcomes and decreased adverse effects.

## 1. Introduction

Inflammatory arthritis is a term used to classify a group of conditions such as rheumatoid arthritis (RA), which affects the immune system and causes pain, stiffness, and joint damage [1,2]. Different therapeutic strategies for decreasing the pain and improving the outcomes of both joint damage and disability caused by RA have rapidly advanced in the past few years.

TNF α has an important role in the pathogenesis of RA, since it is overexpressed in the synovial joints of patients [3]. Therefore, anti-TNF α monoclonal antibody therapies have been pursued to target TNF α and treat RA. These therapies have been effective in most treated RA patients. However, there is a percentage of patients that either fail to respond to the therapy or experience adverse effects from it [4]. Traditional drugs including non-steroidal inflammatory drugs (NSAIDs), glucocorticoids (GCs), disease modifying anti-rheumatic drugs (DMARDs) and biological agents can relieve the pain and control inflammation [5]. However, current treatments are not fully effective and frequently involve high dosage or repeated administration in order to have a therapeutic effect, which can result in a decrease in overall efficacy and patient compliance and cause severe side effects [6]. Thus, to overcome existing limitations, new and advanced therapies designed to tackle inflammatory arthritis are strongly required [7,8].

Nanotechnology, as nanoparticle-based delivery systems, provides a unique series of advantages for the development of delivery systems including high loading capacity, controlled release, prolonged circulation, and target delivery [9]. Even so, these systems have demonstrated unpredictable drug release depending on their polymer structure, production conditions, and particle size [10]. In order to tune drug release, nanoparticles have been combined with different biomaterials [11,12,13,14]. Amongst the studied biomaterials, hydrogels appear to demonstrate the most attractive outcomes. Hydrogels are a particularly appealing type of delivery system. In fact, they can provide the controlled release of nanoparticles over time, offer controllable degradability, and have the capacity to protect loaded drugs from degradation [15,16].

Despite being relatively simple and effective, is has been challenging to translate such approaches into clinical settings. One of the reasons for this may be a reliance on the settings selected for their study. Current in vitro cellular methods, applied in several areas of drug discovery, comprise the use of static conditions which do not emulate the dynamic natural physiological environment found in the human body, thereby failing to produce reliable data [17,18]. Thus, new dynamic methods need to be established to more precisely assess the effectiveness of drugs in in vivo systems. Bioartificial devices, as flow bioreactors, are highly promising tools for tissue engineering and regenerative medicine applications [19]. They can be used for cell culture, therapeutic approaches, and for in vitro organ modeling, offering a more physiologically appropriate environment compared to traditional static conditions [20,21]. Current works have shown that the use of bioreactors to maintain the culture of cell-laden scaffolds enables the proper flow of nutrients and increased the diffusion of oxygen through the 3D structures, thereby mimicking specific aspects of native tissues [21,22].

In previous works, Tyramine-Gellan Gum (Ty-GG) hydrogels [23] and Tyramine-Gellan Gum/Silk Fibroin (Ty-GG/SF) hydrogels [24] showed good resistance to degradation, high mechanical strength, and a suitable drug controlled release for treating inflammatory conditions such as RA. Monoclonal anti-TNF α antibody (anti-TNF α Ab) linked to chondroitin sulfate (CS) modified poly(amidoamine) (CS/PAMAM) dendrimer nanoparticles (NPs) were developed and demonstrated a more targeted and effective therapeutic effect for the management of RA [25]. Herein, with the aforementioned in mind, it was hypothesized that by combining the two approaches, an improved delivery system would be achieved with a sustained release that would last for longer periods. For so, a system based on anti-TNF α Ab-CS/PAMAM dendrimer NPs loaded into Ty-GG and Ty-GG/ SF hydrogels was developed as a promising drug delivery vehicle with improved therapeutic efficacy for the treatment of RA. The evaluation of the developed system was performed in a human monocytic cell line (THP-1)-based inflammation in vitro model under both static and dynamic culture conditions.

## 2. Materials and Methods

### 2.1. Anti-TNF α Ab-CS/PAMAM Dendrimer NPs

#### 2.1.1. Functionalization

Mouse monoclonal antibody [2C8] (26,000 g mol^−1^) (ab8348, Abcam, Cambridge, UK) was linked to CS/PAMAM dendrimer NPs previously produced by the authors [25]. The linkage was obtained through a typical carbodiimide chemistry reaction. Briefly, a 2 g L^−1^ solution of CS/PAMAM in 19.52 g L^−1^ of 2-N-Morpholinoethanesulfonic acid hydrate (MES) (Sigma-Aldrich, St. Louis, MO, USA) was mixed with *N*-(3-Dimethylaminopropyl)-*N*′-ethylcarbodiimide hydrochloride (EDC) (Sigma-Aldrich, St. Louis, MO, USA) (4 equivalents) and NHS sulfo-NHS *N*-Hydroxysulfosuccinimide sodium salt ≥98% (HPLC) (Sigma-Aldrich, St. Louis, MO, USA) (two equivalents) for 15 min. At the same time, 20 equivalents of anti-TNF α Ab were added with EDC (four equivalents) and were stirred for 15 min. Then, a CS/PAMAM dendrimer NPs mixture was added to the anti-TNF α mAb mixture and stirred for 24 h. Upon this time, the solution was dialyzed against distilled water for 48 h. Anti-TNF α Ab-CS/ PAMAM dendrimer NPs were obtained by freezing the solution at −80 °C and freeze-drying (Telstar-LyoAlfa 10/15) up to 48 h.

#### 2.1.2. Evaluation of Anti-TNF α Ab Conjugation Efficacy and Retention of Biological Activity

The efficacy of the modification was assessed by fluorescence spectrometer FP-8500 (Jasco, Oklahoma City, OK, USA). For that, anti-TNF α Ab-CS/PAMAM dendrimer NPs and CS/PAMAM dendrimer NPs were allowed to react with a secondary antibody Alexa Fluor 488 rabbit anti-mouse lgG (H + L) (Molecular Probes, Eugene, OR, USA) in PBS solution (1:1000) for 1 h. Then, the solutions were analyzed in the fluorescence spectrometer.

The capacity of anti-TNF α Ab-CS/PAMAM dendrimer NPs to capture TNF α was also evaluated. For the evaluation, modified dendrimers were incubated with 1000 pg mL^−1^ of TNF α (Peprotech, London, UK) for 4 h at room temperature. As controls, non-modified dendrimers were also incubated. After the time of reaction, the solutions were centrifuged, and the supernatants were kept at −80 °C for further analysis. The concentration of free TNF-α in the supernatant samples was determined using a Human TNF-alpha DuoSET ELISA kit (R&D Systems, Minneapolis, MN, USA) and a DuoSet Ancillary Reagent Kit 2 (R&D Systems, Minneapolis, MN, USA), following the manufacturer’s instructions. The samples were measured at an optical density of 450 nm using a microplate reader (Synergy HT, BIO-TEK, Winooski, VT, USA). Absorbance values were converted into concentrations using a standard curve of TNF-α in the range of 0 to 1000 pg mL^−1^.

#### 2.1.3. Production of Anti-TNF α Ab-CS/PAMAM Dendrimer NPs Loaded Ty-GG and Ty-GG/SF Hydrogels

Ty-GG previously produced by the authors [23] was used to obtain the investigated hydrogels. With respect to the Ty-GG/SF hydrogels, silk fibroin [24] was extracted from silkworm cocoons (Portuguese Association of Parents and Friends of Mentally Disabled Citizens (APPACDM, Castelo Branco, Portugal)). Briefly, the cocoons were cut into fragments and boiled for 30 min in a 0.02 M Sodium carbonate solution (Laborspirit, Loures, Portugal) to remove sericin. Boiled silkworm cocoons were washed with distilled water and dried at 70 °C. The dry silkworm cocoons were then dissolved in the oven with 9.3 M Lithium bromide (Laborspirit, Loures, Portugal) for 4 h at 70 °C. The dissolved solution was dialyzed using a dialysis tubing, benzoylated (Laborspirit, Loures, Portugal) for 48 h against distilled water, in order to remove LiBr. Silk fibroin was kept at 4 °C until further use.

To prepare the hydrogels, first a solution of horseradish peroxidase (HRP) (0.84 mg mL^−1^) (Sigma-Aldrich, St. Louis, MO, USA) in PBS and a solution of hydrogen peroxide (H_2_O_2_) (0.36% (*v*/*v*)) (VWR, Radnor, PA, USA) in distilled water were prepared. Then, 1% Ty-GG solution (*w*/*v*) was prepared in distilled water and used for the preparation of Ty-GG hydrogels and Ty-GG/SF hydrogels. To obtain the Ty-GG/SF hydrogels, 1% (*w*/*v*) of Ty-GG solution was mixed with 2% (*w*/*v*) SF (1:1) solution. At this point, anti-TNF α Ab-CS/PAMAM dendrimer NPs were mixed in the Ty-GG and Ty-GG/SF solutions at a final concentration of 0.5 mg mL^−1^. Then, two different crosslinking levels were tested by adding different amounts of HRP and H_2_O_2_ solutions to Ty-GG and Ty-GG/SF solutions, hereafter denominated C1, C2, C3, and C4, as described in Table 1. Each condition without anti-TNF α Ab-CS/PAMAM dendrimer NPs were used as control (hereafter designated C1 CTRL, C2 CTRL, C3 CTRL, and C4 CTRL). Then, the mixtures were transferred into polypropylene molds and incubate at 37 °C until complete gelation.

#### 2.1.4. Distribution of FITC-CS/PAMAM Dendrimer NPs throughout Ty-GG and Ty-GG/SF Hydrogels

To assess the distribution profile of CS/PAMAM dendrimer NPs within Ty-GG and Ty-GG/SF hydrogels, the nanoparticles were labelled with Fluorescein isothiocyanate (FITC) (Sigma-Aldrich, St. Louis, MO, USA). Initially, 10 mg mL^−1^ of CS/PAMAM dendrimer NPs solution was prepared in a carbonate-bicarbonate coupled buffer (pH 9.2). Then, 50 µL of 10 mg mL^−1^ of FITC in anhydrous dimethyl sulfoxide (DMSO) (VWR, Radnor, PA, USA) solution was added per each mL of CS/PAMAM dendrimer NPs solution under agitation and kept in the dark at 4 °C for 8 h. In the end, the FITC-labelled CS/PAMAM dendrimer NPs were dialyzed in ultrapure water for 48 h and freeze-dried. Then, FITC-CS/PAMAM dendrimer NPs, at a final concentration of 0.1 mg mL^−1^, were mixed with Ty-GG and Ty-GG/SF solutions and HRP and H_2_O_2_ solutions (as described in Table 1). After crosslinking, the hydrogels were immersed in PBS and kept in a water bath at 37 °C for 24 h to remove free FITC. The hydrogels were observed under a confocal microscope (Leica TCS SP8).

#### 2.1.5. Release Profile of Anti-TNF α Ab-CS/PAMAM Dendrimer NPs from Ty-GG and Ty-GG/SF Hydrogels

To evaluate the release profile of anti-TNF α Ab-CS/PAMAM dendrimer NPs, each release system developed, C1, C2, C3, and C4 (more details in Table 1), were immersed in PBS at 37 °C. After 3 h, 24 h, 72 h, 168 h, 336 h, and 504 h, the PBS solution which was in contact with hydrogels was collected to an Eppendorf 1.5 mL and kept at −80 °C until further analysis. To formulate the calibration curve, anti-TNF α Ab-CS/PAMAM dendrimer NPs dilutions were prepared ranging from 0 mg mL^−1^ to 0.5 mg mL^−1^. The UV absorbance of dendrimers NPs was read at 280 nm in a microplate reader to quantify the anti-TNF α Ab-CS/PAMAM dendrimer release (EMax; Molecular Devices, Sunnyvale, CA, USA). Three samples per condition were evaluated at each time point. The absorbance values were converted into concentrations using the calibration curve.

### 2.2. THP-1 Cells-Based Inflammation In Vitro Model: Static Conditions

#### 2.2.1. Cell Culture

The Human monocytic cell line (THP-1) (SIGMA, USA) was expanded in RPMI 1640 Medium, GlutaMAX™ Supplement, HEPES (Thermo Fisher Scientific, Waltham, MA, USA), supplemented with 10% fetal bovine serum (PAA; Pasching, Austria) and 1% (*v/v*) of penicillin and streptomycin (Gibco, Life Technologies, Grand Island, NY, USA), under standard culture conditions (i.e., at 37 °C in a humidified atmosphere containing 5 vol% CO_2_). When ≈80% confluence was reached, cells were trypsinized and seeded in a well of a 24-well plate at a density of 5 × 10^5^ cells per well. For induction of THP-1 cells’ differentiation into macrophages, cells were cultured under RPMI with 100 nM phorbol 12-myristate-13-acetate (PMA) (Sigma-Aldrich, St. Louis, MO, USA), hereafter designated as healthy cells. After 24 h, the medium was replaced with a RPMI medium without PMA and incubated for another 48 h. At this point, to develop an inflammation in vitro model, cells were incubated with 100 ng mL^−1^ of Lipopolysaccharide (LPS) (Sigma-Aldrich, St. Louis, MO, USA) in an RPMI medium and incubated for 5 h to induce an inflammatory response.

Cells under LPS stimulation were incubated with each release system developed, C1, C2, C3, and C4 (more details in Table 1), respective controls (C1 CTRL, C2 CTRL, C3 CTRL, and C4 CTRL) and 0.5 mg mL^−1^ anti-TNF α Ab-CS/PAMAM dendrimer NPs. Only cells under LPS stimulation were studied (hereafter designated as LPS stimulation). Cells’ metabolic activity and proliferation were monitored along seven days of culture. The TNF α neutralization was assessed along 14 days of culture.

#### 2.2.2. Cells’ Metabolic Activity

Cells’ metabolic activity was evaluated on days one, three, and seven of culture with Alamar Blue at each time point. For this, an RPMI culture medium containing 10% (*v*/*v*) of Alamar Blue^®^ (BioRad, Oxford, UK) was added to the different conditions.

The culture plates were kept in the dark, at 37 °C in the CO_2_ incubator for 4 h. Afterward, 100 µL of each well were transferred in triplicate to 96-well plates. The fluorescence was read at an excitation wavelength of 530/25 nm and an emission wavelength of 590/535 nm, using a microplate reader (Synergy HT, BioTek, Instruments, Winooski, VT, USA).

#### 2.2.3. Cells’ Proliferation

The proliferation of the THP-1 cells at days one, three, and seven of culture was analyzed by dsDNA quantification. At each timepoint, cells were washed with PBS solution and lysed with ultrapure water. The cells’ lysate solution was placed into 1.5 mL microtubes and then stored at −80 °C for further analysis. The Quanti-IT PicoGreen dsDNA Assay Kit (Alfagene, Lisboa, Portugal) was used to quantify dsDNA, accordingly with manufacturers’ instructions. Briefly, 28.7 µL of each sample was mixed with 71.3 µL of PicoGreen solution and 100 µL 1X TE buffer in a well of a 96-well white microplate. Then, the plate was incubated in the dark for 10 min and the fluorescence was read using an excitation of 480/20 nm and an emission of 528/20 nm in a microplate reader. DNA concentration was determined using a standard curve in the range of 2 to 0 µL mL^−1^.

#### 2.2.4. Assessment of TNF α Neutralization

The neutralization of TNF α as evaluated at one, three, seven and fourteen days of culture. At these time-points, the medium of each condition was recovered and stored at −80 °C. To determine the levels of TNF α captured, the TNF-α concentration in the samples was measured using the Human TNF-alpha DuoSET ELISA kit and the DuoSet Ancillary Reagent Kit 2, following manufacturers’ instruction. The optical density was measured at 450 nm using the microplate reader and the values were converted into concentration using a TNF-α calibration curve ranging from 1000 to 0 pg mL^−1^.

### 2.3. THP-1 Cells-Based Inflammation In Vitro Model: Dynamic Conditions

#### 2.3.1. Cell Culture in a Dual-Chamber Bioreactor

THP-1 cells were seeded at a density of 5 × 10^5^ cells/well in TCP coverslips in 24-well plates. As conducted under static conditions, for induction of THP-1 cell differentiation, cells were cultured under RPMI with 100 nM PMA. After 24 h, the medium was replaced with RPMI medium without PMA and incubated for another 48 h. Then, the cells were incubated with 100 ng mL^−1^ of LPS in an RPMI medium and incubated for 5 h to induce an inflammatory response. After LPS stimulation, the coverslips were transferred to the lower chamber of a dual-chamber bioreactor [26] with an RPMI medium contained LPS and the therapeutic effect of developed release systems was evaluated.

#### 2.3.2. Therapeutic Effect of Developed Release Systems

To evaluate the therapeutic effect of the developed approaches under dynamic conditions, one condition of each delivery system was selected and added to the cells cultured on the lower chamber of the bioreactor. Then, the entire piping system was assembled and the syringes were filled with medium and connected to the syringe pump. The dual-chamber bioreactor was kept at 37 °C in a humidified 5% CO_2_ atmosphere and the compartment containing the cells and hydrogels was perfused at a rate of 12.5 µL h^−1^. After one, three, seven, and fourteen days of perfusion, the culture medium contained in the collection tubes was removed and stored at −80 °C to analyze of the amount of TNF α present through an ELISA assay, as described above. Delivery systems cultured in THP-1 cell-based inflammation in vitro models under standard static conditions were used as control.

### 2.4. Statistical Analysis

Statistical analysis was performed by GraphPad Prism 8 version, (GraphPad Software Inc, San Diego, CA, USA) where a Shapiro–Wilk normality test was previously made to evaluate the data normality. Statistical significances were obtained as * *p* < 0.05. All assays were triplicated and the results were presented as mean ± standard deviation.

## 3. Results and Discussion

The present work is focused on the development of anti-TNF α Ab-CS/PAMAM dendrimer NPs loaded into Ty-GG and Ty-GG/ SF hydrogels as a promising delivery system. This way, hydrogels would increase the controlled and sustained release of anti-TNF α Ab-CS/PAMAM dendrimer NPs at the target site, improving their therapeutic efficacy while requiring less frequent administrations [15]. With this in mind, the developed NPs loaded-hydrogels were evaluated in a THP-1 cells-based inflammation in vitro model (Figure S1) under static standard conditions and dynamic conditions using a bioreactor.

### 3.1. Evaluation of Anti-TNF α Ab Conjugation and TNF α Sequestration Efficacy 

**Figure 1:**
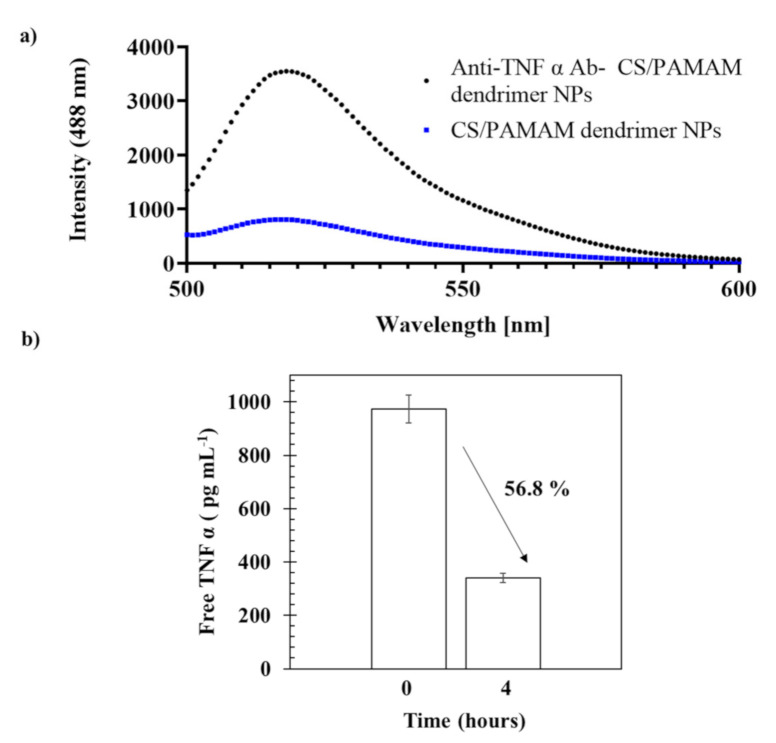
Anti-TNF α antibody conjugation to CS/PAMAM dendrimer NPs. (**a**) Fluorescence spectroscopy of CS/PAMAM dendrimer NPs and anti-TNF α Ab-CS/PAMAM dendrimer NPs. (**b**) Percentage of TNF α captured by anti-TNF α Ab-CS/PAMAM dendrimer NPs. Data shown as Mean ± SD.

Free antibodies have been reported to present the capacity to neutralize the target agent, TNF α [27]. Herein, we aimed to investigate the biological activity of anti-TNF α conjugated CS/PAMAM dendrimer NPs in vitro. A monoclonal anti-TNF α antibody was linked to CS/PAMAM dendrimer NPs and the efficacy of the linking reaction, as well as the preservation of its biological activity (i.e., its ability to capture TNF α) were evaluated (Figure 1). Fluorescence spectroscopy was used to assess the success of conjugation (Figure 1a) with both previous conjugation of the CS/PAMAM dendrimer NPs (both with and without linked Ab) and a secondary antibody labeled with Alexa Fluor 488 dye. The intensity of the emission spectra of Alexa 488 between 500 and 600 nm for the anti-TNF α Ab-CS/PAMAM dendrimer NPs was significantly higher (≃3500) than CS/PAMAM dendrimer NPs without anti-TNF α Ab (≃800). Therefore, the conjugation of CS/PAMAM dendrimer NPs with the anti-TNF α Ab was successfully achieved, despite the fact that some unspecific secondary Ab binding was observed (as expected).

To further assess the anti-TNF α Ab-CS/PAMAM dendrimer NPs’ effectiveness in TNF α sequestering, the difference between the initial and the final amount of free TNF α in solution was quantified following the dendrimer NPs’ addition (Figure 1b). The data showed that after 4 h of incubation, the degree of capture was 56.8%. This demonstrates that the immobilization process did not compromise the biological activity of the anti-TNF α Ab, as demonstrated by the retention of its capacity to capture TNF α.

### 3.2. Production of Anti-TNF α Ab-CS/PAMAM Dendrimer NPs Loaded-Ty-GG and Ty-GG/SF Hydrogels: Dendrimer NPs Distribution and Release Profile

**Figure 2:**
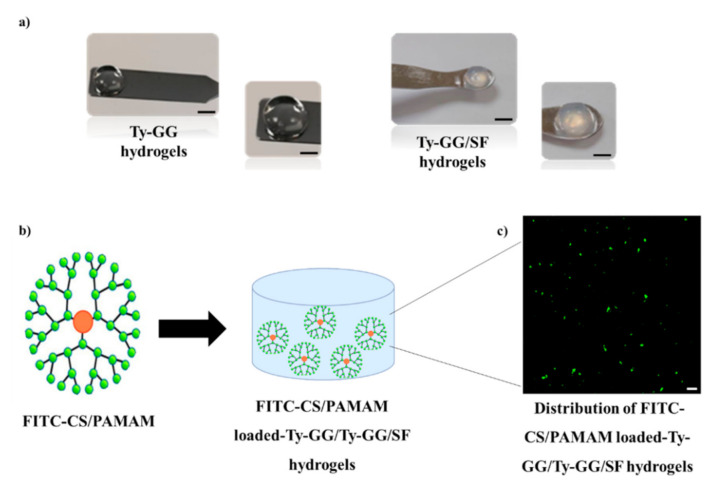
Anti-TNF α Ab-CS/PAMAM dendrimer NPs loaded-Ty-GG and Ty-GG/SF hydrogels. (**a**) Representative images of Ty-GG hydrogels and Ty-GG/SF hydrogels. Scale bar: 10 cm. (**b**) Schematic representation of FITC-CS/PAMAM dendrimer NPs and FITC-CS/PAMAM dendrimer NPs loaded-Ty-GG and Ty-GG/SF hydrogels. Scale bar: 5 μm. (**c**) Representative fluorescence image of FITC-CS/PAMAM dendrimer NPs distributed within Ty-GG and Ty-GG/SF hydrogels, showing the NPs in green.

At this point, anti-TNF α Ab-CS/PAMAM dendrimer NPs were loaded into Ty-GG and Ty-GG/SF disc hydrogels (Figure 2). The gelation of Ty-GG and Ty-GG/SF solutions within polypropylene molds produced discs with similar sizes. However, it was clear that the addition of SF resulted in more opaque hydrogels (Figure 2a). To assess the distribution of dendrimer NPs throughout the hydrogels, they were labelled with FITC and observed using confocal microscopy (Figure 2b). It was possible to observe that CS/PAMAM dendrimers NPs were uniformly dispersed across the hydrogel network, exhibiting low aggregation as expected (since the encapsulation of NPs in hydrogels reduces particles’ aggregation) [28]. This dispersion uniformity guarantees a larger exposure of the surface of the nanoparticle and, consequently, better performance as a therapeutic agent [29].

**Figure 3:**
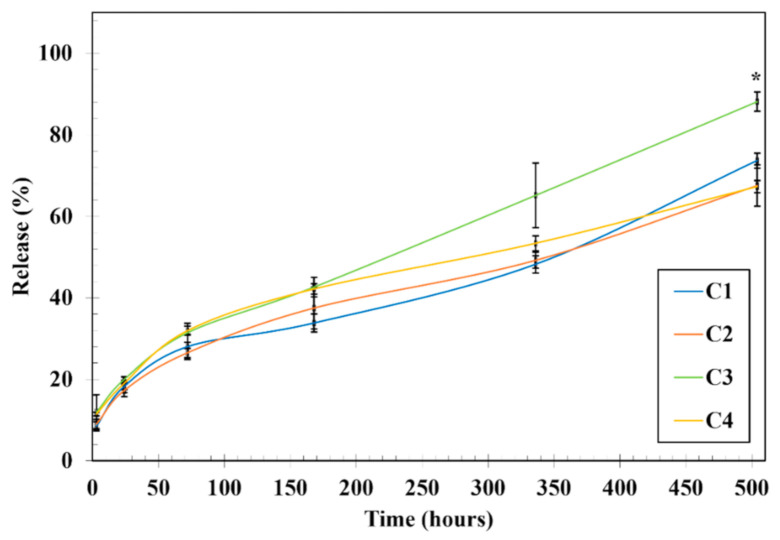
Release profile of anti-TNF α Ab-CS/ PAMAM dendrimer NPs. Release profile of anti-TNF α Ab-CS/ PAMAM dendrimer NPs from Ty-GG (C1 and C2) and Ty-GG/SF hydrogels (C3 and C4) with different crosslinking levels after 3, 24, 48, 72, 168, 336, and 504 h. Data shown as Mean ± SD. * indicates significant differences when comparing C3 and C4 at time point 504 h.

Considering the success of the hydrogel-based NPs’ encapsulation, it was critical to optimize Ty-GG and Ty-GG/SF hydrogels’ crosslinking levels to maximize anti-TNF α Ab-CS/PAMAM dendrimer NPs (Figure 3). Thus, anti-TNF α Ab-CS/PAMAM dendrimer NPs’ release profile from hydrogels network was assessed over time. Ty-GG hydrogels, denominated C1 (more details at Table 1), released 73.65% ± 1.85% of the NP’s, while the same hydrogels but with higher amounts of crosslinking (C2) released 67.12% ± 4.03%, over 21 days. A similar profile was observed for Ty-GG/SF hydrogels; C3 condition released 88.13% ± 2.36%, while the condition with higher crosslinking levels, C4, released 67.28% ± 1.47% of the encapsulated NPs over 21 days. When analyzing the conditions with lower levels of crosslinking (C1 and C3), Ty-GG/SF hydrogels showed a faster release when compared to Ty-GG hydrogels. However, when comparing the conditions with higher crosslinking levels (i.e., C2 and C4) no statistical differences were observed among the conditions. These conditions presented a slower release of the dendrimer NPs with the increase of crosslinking level, possibly due the contraction of the hydrogel mesh. In this sense, C2 and C4 showed a more suitable drug release profile when compared to C1 and C3. The slower drug release profile may allow a longer therapy to the patient and, consequently, a less frequent administration, thereby reducing the side effects caused by drug overdose. Noteworthy, none of the conditions reached a maximum drug release after 21 days of evaluation. Considering the results obtained, it was possible to verify that hydrogels can be fine-tuned to present a controlled drug release profile over time. This can allow a prolonged therapeutic effect of anti-TNF α Ab. In this line of reasoning, less frequent administration of drugs would be necessary and would improve the quality of life of patients.

### 3.3. Evaluation of Anti-TNF α Ab-CS/ PAMAM Dendrimer NPs Loaded-Ty-GG and Ty-GG/SF Hydrogels Effects on THP-1 Cells-Based Inflammation In Vitro Models: Static Conditions

**Figure 4:**
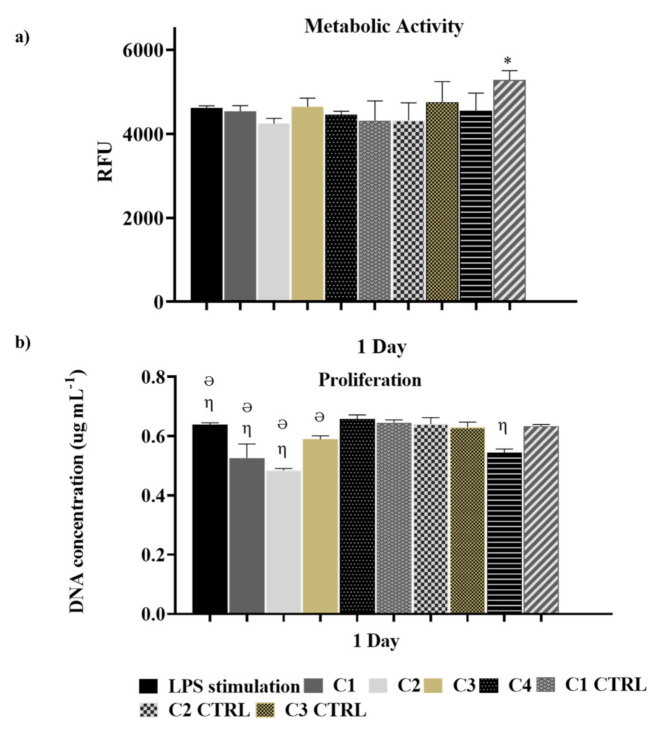
Evaluation of anti-TNF α Ab-CS/ PAMAM dendrimer NPs loaded-Ty-GG and Ty-GG/SF hydrogels effects on cells metabolic activity and proliferation. (**a**) THP-1 cells’ metabolic activity upon culture with anti-TNF α Ab-CS/ PAMAM dendrimer NPs loaded-Ty-GG (C1 and C2) and Ty-GG/SF (C3 and C4) hydrogels and controls for 1 day of culture. (* indicates significant differences when comparing Dendrimer NPs with LPS stimulation at time point 1 day); and (**b**) THP-1 cells’ proliferation by DNA quantification of THP-1 cells upon culture with anti-TNF α Ab-CS/ PAMAM dendrimer NPs loaded-Ty-GG (C1 and C2) and Ty-GG/SF (C3 and C4) hydrogels and controls for 1 day of culture. (η indicates significant differences when comparing with LPS stimulation. Ə indicates significant differences when comparing with respective CTRL). Data shown as Mean ± SD.

In RA, there is a disproportion between pro-inflammatory and anti-inflammatory cytokines, resulting in a more pronounced pro-inflammatory phenotype [30,31,32,33]. For this reason, most therapies developed to decrease inflammation by inhibiting pro-inflammatory cytokines are evaluated using cell inflammation in vitro models [7,34]. For this reason, the influence of the different conditions of anti-TNF α Ab-CS/ PAMAM dendrimer NPs loaded-Ty-GG (C1 and C2) and Ty-GG/SF (C3 and C4) hydrogels were evaluated using an inflammation in vitro model created by the induction of THP-1 cells. Ty-GG and Ty-GG/SF hydrogels without dendrimer NPs (C1 CTRL, C2 CTRL, C3 CTRL, and C4 CTRL) and dendrimer NPs were used as controls. First, the effect of loaded hydrogels on LPS stimulated THP-1 cells’ metabolic activity and proliferation was quantified for seven days using Alamar blue and DNA quantification assays, respectively (Figure 4). From day one up to day seven, THP-1 cells, under all of the different conditions of Ty-GG and Ty-GG/SF hydrogels, were shown to be metabolically active over time. Hydrogels missing dendrimer NPs represent the only instance wherein there was higher metabolic activity at the first time point (24 h) when compared with the LPS stimulated cells. The enhanced metabolic activity which was observed can be explained by the increase of energy necessary for the internalization of dendrimers. In fact, considering the conditions that contained dendrimer NPs, they were released in a controlled and sustainable way from the hydrogels. While in this condition, dendrimer NPs were easily accessible, being internalized at a higher rate. At three and seven days, there was an increase of metabolic activity in all conditions; no statistical differences were observed among the various conditions.

DNA quantification was used to assess THP-1 cells’ proliferation under different conditions. At day one, cell proliferation was decreased in C1, C2, C3, and C4 CTRL as compared to the LPS stimulated cells. However, this effect dissipated over seven days, with no final differences among the conditions. The lack of influence of the GG hydrogels and dendrimer nanoparticles over cell viability has been previously known for over a decade [35,36].

Overall, the results of metabolic activity and cell proliferation analysis provided evidence regarding the safety of these materials for biomedical applications.

**Figure 5:**
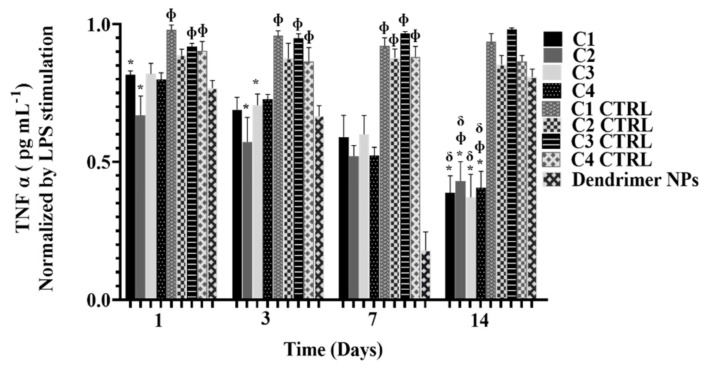
Quantification of TNF α free in the culture medium along 14 days of culture. Results were normalized by the TNF α values obtained in cultures stimulated by LPS. Data shown as Mean ± SD. * indicates significant differences when comparing with respective CTRL. Φ indicates significant differences when comparing with Dendrimer NPs at each time-point. δ indicates significant differences when comparing with day one.

In order to evaluate the capturing efficacy of TNF α by C1, C2, C3, and C4, the level of free TNF α in the culture medium along 14 days of culture was quantified (Figure 5). Again, C1 CTRL, C2 CTTRL, C3 CTRL, C4 CTRL, and dendrimer NPs were analyzed. Although at the beginning of culture only C1 and C2 showed significant differences from their respective controls in the neutralization of free TNF α, after 14 days all the conditions (C1, C2, C3, and C4) were shown to significantly neutralize free TNF α. The neutralization levels at day 14 were also significantly different from day one for all the studied conditions. Furthermore, the results are in agreement with those obtained from the drug release studies. C1 and C3 (lower amount of crosslinking) showed a faster release profile. Moreover, the degree of TNF α capture was higher compared to C2 and C4 (i.e., higher amounts of crosslinking) where the release profile was slower and, therefore, the degree of neutralization was lower. Free dendrimer NPs neutralized higher amounts of TNF α comparing with controls since day one, as expected, reaching its maximum at day seven similarly to the sequestration achieved by the anti-TNF α Ab-CS/PAMAM dendrimer NPs. However, at day 14, levels of free TNF α abruptly increased using free dendrimer NPs, while NPs-loaded hydrogels retained their capacity to neutralize TNF α. Noteworthy, dendrimer NPs at day 14 showed levels of free TNF α similar to the controls, corroborating dendrimer NPs’ low long-term effect. These results showcase the advantages of NP-loaded hydrogels in drug delivery to chronic diseases, such as RA, considering the prolonged and continuous effect of the therapy. Hence, this approach may allow the reduction of side effects, providing a better quality of life to the patient. These data showed the potential of Gellan Gum and SF as drug delivery systems and corroborates other previous studies [37,38].

### 3.4. Evaluation of Anti-TNF α Ab-CS/ PAMAM Dendrimer NPs Loaded-Ty-GG and Ty-GG/SF Hydrogels Effects on THP-1 Cell-Based Inflammation In Vitro Models: Dynamic Conditions

**Figure 6:**
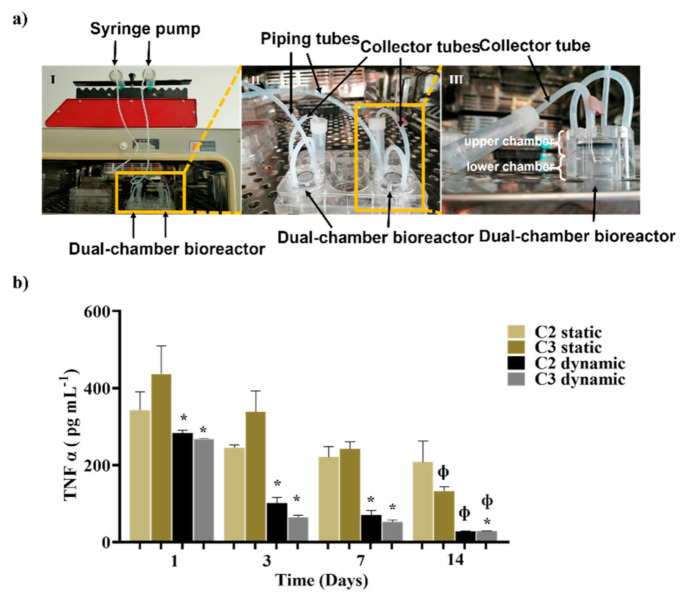
Evaluation of anti-TNF α Ab-CS/ PAMAM dendrimer NPs loaded-Ty-GG and Ty-GG/SF hydrogels effects under dynamic conditions. (**a**) Representative images of Dynamic culture of THP-1 cell-based inflammation in vitro model using a dual-chamber bioreactor showing the (I) dual-chamber bioreactor connected to a syringe pump; (II) higher amplification of the bioreactor inserted in a 6-well plate showing the piping tubes and collector tubes; and (III) enlarged image of the dual-chamber bioreactor where it is possible to see the upper and lower chamber. (**b**) Amount of TNF α present in the medium, in contact with C2 and C3 in static and dynamic conditions. Data shown as Mean ± SD. * indicates significant differences when comparing dynamic conditions with static conditions, Φ indicates significant differences when comparing with day 1.

A dual-chamber bioreactor was used to mimic the dynamic physiological environment found in the human cartilage and obtain more reliable data concerning the challenges faced by the drug delivery system. Conditions that led to faster and slower NPs release were chosen for this setup. C3 was the condition that presented higher NPs release levels and a better ability to decrease free TNF α. Since C3 is a Ty-GG/SF hydrogel-based strategy and C2 and C4 were conditions with similarly slow-release profiles, C2 was chosen (considering it is based on a Ty-GG hydrogel). Therefore, anti-TNF α Ab-CS/ PAMAM dendrimer NPs loaded-Ty-GG and Ty-GG/SF hydrogels were tested for 14 days using a THP-1 cell-based inflammation in vitro model under dynamic conditions (Figure 6). The control comprised THP-1 cell-based inflammation in vitro models under standard static conditions.

It was possible to verify that the TNF α concentration present in the supernatant medium significantly decreased along the 14 days of culture under both dynamic and control conditions. However, under static conditions, C2 was the only one presenting no differences to the control, in accordance with the previously release profile evaluation. Firstly, analyzing the results obtained for static and dynamic conditions, it was observed that the concentration of TNF α was always lower in dynamic conditions. However, in dynamic conditions, on day 14, the levels of free TNF α decreased abruptly, indicating that all anti-TNF α Ab-CS/ PAMAM dendrimer NPs were released from the hydrogels. Under static conditions, a less accentuated decrease of TNF α was observed, indicating a slower release of dendrimer NPs over time. Noteworthy, no significant differences were observed between the dendrimer NP-loaded hydrogels tested.

## 4. Conclusions

In this study, an improved system based on anti-TNF α Ab-CS/PAMAM dendrimer NPs loaded into Ty-GG and Ty-GG/SF hydrogels to tackle inflammatory diseases such as RA was developed. The produced anti-TNF α Ab- CS/PAMAM dendrimer NPs demonstrated a capacity to maintain Ab biological activity, enabling a high immobilization degree of TNF α. Furthermore, it was possible to fine-tune anti-TNF α Ab-CS/PAMAM dendrimer NP-loaded Ty-GG and Ty-GG/SF hydrogels by altering crosslinking levels, thereby enabling the control of the dendrimer NPs release profile over time. The in vitro studies conducted using the THP-1 cell-based inflammation model under static conditions demonstrated that cells cultured with the developed dendrimer NPs-loaded hydrogels were metabolically active and proliferative along the culture time. When their capacity to capture TNF α was evaluated, it was observed that developed systems retained their capacity to neutralize TNF α even after 14 days. Finally, dynamic studies performed with the THP-1 cell-based inflammation model showed that the dendrimer NP-loaded hydrogels maintained an even higher therapeutic effect over time as compared with the static studies, demonstrating the importance of using a physiologically mimetic environment. These relevant results support the successful use of these drug delivery systems in several inflammatory diseases, including RA, in in vivo settings. Overall, the developed approaches which make use of bioengineered nanoparticle-loaded hydrogels can fill the current gaps concerning traditional therapies, providing a more targeted and thus more effective treatment while enabling less frequent administration and, thus, reducing the side effects caused by an overdosing of drugs.

The developed approach showed potential to be used as personalized medicine to obtain better therapeutic outcomes and decreased adverse effects. Thus, in the near future, it is expected that the knowledge acquired in the tissue engineering field can be an important tool in the development of clinical medicine.

## Figures and Tables

**Table 1:** Anti-TNF α Ab-CS/PAMAM loaded-Ty-GG and Ty-GG/SF hydrogels with different crosslinking levels.

Designation	Ty-GG (1% *w*/*v*)	SF (2% *w*/*v*)	Dendrimer NPs (1 mg mL^−1^)	HRP (0.84 mg mL^−1^)	H_2_O_2_ (0.36% *v/v*)
C1	167 µL	-	97 µL	16.6 µL	10.83 µL
C2	167 µL	-	100 µL	18.3 µL	15 µL
C3	83.5 µL	83.5 µL	97 µL	16.6 µL	10.83 µL
C4	83.5 µL	83.5 µL	100 µL	20 µL	13.3 µL

## Supplementary Materials

The following are available online at https://www.mdpi.com/article/10.3390/pharmaceutics13081111/s1, Figure S1. Amount of free TNF α in medium in THP-1 cells culture without LPS stimulation, designated healthy cells, and with LPS stimulation after 1 day, confirming the successful development of THP-1 cells-based inflammation in vitro model.
