# Supplementary material for: Bioengineered Nanoparticles Loaded-Hydrogels to Target TNF Alpha in Inflammatory Diseases
Source: Pharmaceutics. 2021 Jul 21;13(8):1111. doi: 10.3390/pharmaceutics13081111 (PMC8400713; doi:10.3390/pharmaceutics13081111)
Supplement: Supplementary file 1 [file pharmaceutics-13-01111-s001.zip › pharmaceutics-1267307-supplementary.pdf]

pharmaceutics

Supplementary Materials: Bioengineered Nanoparticles Loaded-Hydrogels to Target TNF Alpha in Inflammatory Diseases

Isabel Matos Oliveira, Diogo Castro Fernandes, Fátima Raquel Maia, Raphael Faustino Canadas, Rui Luís Reis and Joaquim Miguel Oliveira

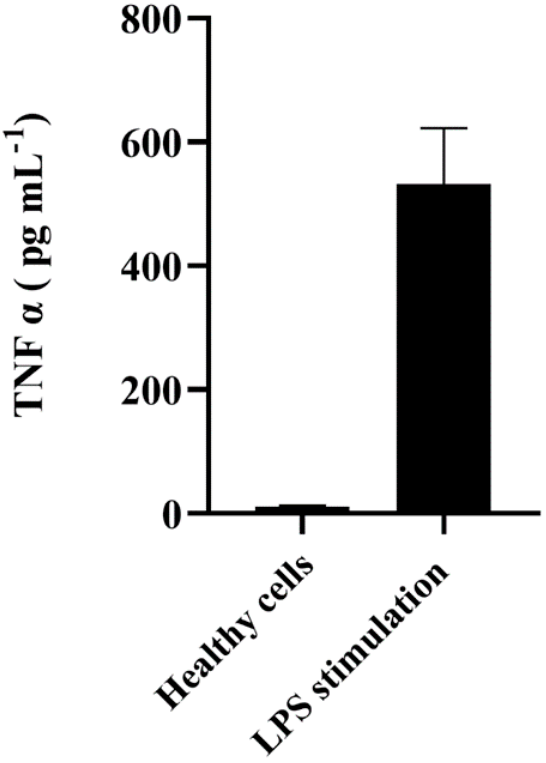

Figure S1. Amount of free TNF α in medium in THP-1 cells culture without LPS stimulation, designated healthy cells, and with LPS stimulation after 1 day, confirming the successful development of THP-1 cells-based inflammation in vitro model.
